# Designing a Candidate Multi-Epitope Vaccine against Transmissible Gastroenteritis Virus Based on Immunoinformatic and Molecular Dynamics

**DOI:** 10.3390/ijms25168828

**Published:** 2024-08-13

**Authors:** Yihan Bai, Mingxia Zhou, Naidong Wang, Yi Yang, Dongliang Wang

**Affiliations:** 1 College of Biology, Hunan University, Changsha 410082, China; byh248950700@stu.hunau.edu.cn; 2 Hunan Provincial Key Laboratory of Protein Engineering in Animal Vaccines, College of Veterinary Medicine, Hunan Agricultural University, Changsha 410128, China; mingxiazhou@stu.hunau.edu.cn (M.Z.); naidongwang@hunau.edu.cn (N.W.); yiyang@hunau.edu.cn (Y.Y.)

**Keywords:** TGEV, spike glycoprotein, molecular docking, epitope

## Abstract

Transmissible gastroenteritis virus (TGEV) is an etiological agent of enteric disease that results in high mortality rates in piglets. The economic impact of the virus is considerable, causing significant losses to the pig industry. The development of an efficacious subunit vaccine to provide promising protection against TGEV is of the utmost importance. The viral antigen, spike glycoprotein (S), is widely regarded as one of the most effective antigenic components for vaccine research. In this study, we employed immunoinformatics and molecular dynamics approaches to develop an ‘ideal’ multi-epitope vaccine. Firstly, the dominant, non-toxic, highly antigenic T (Th, CTL) and B cell epitopes predicted from the TGEV S protein were artificially engineered in tandem to design candidate subunit vaccines. Molecular docking and dynamic simulation results demonstrate that it exhibits robust interactions with toll-like receptor 4 (TLR4). Of particular significance was the finding that the vaccine was capable of triggering an immune response in mammals, as evidenced by the immune simulation results. The humoral aspect is typified by elevated levels of IgG and IgM, whereas the cellular immune aspect is capable of eliciting the robust production of interleukins and cytokines (IFN-γ and IL-2). Furthermore, the adoption of *E. coli* expression systems for the preparation of vaccines will also result in cost savings. This study offers logical guidelines for the development of a secure and efficacious subunit vaccine against TGEV, in addition to providing a novel theoretical foundation and strategy to prevent associated CoV infections.

## 1. Introduction

Coronavirus (CoV) is a member of the family *Coronaviridae* and the subclass *Nidoviridae*. One of its characteristics is its ability to infect diverse hosts, including humans and other animals, and cause illness in these hosts. In recent years, pandemics of severe acute respiratory syndrome (SARS), Middle East respiratory syndrome (MERS), and coronavirus disease 2019 (COVID-19), caused by distinct members of the coronavirus family, have presented a significant risk to human and animal health as well as environmental and economic development globally [1]. To date, coronaviruses (CoVs) have been classified into four genera, including *Alphacoronavirus*, *Betacoronavirus*, *Gammacoronavirus*, and *Deltacoronavirus*. In *Alphacoronavirus*, the transmissible gastroenteritis virus (TGEV) has been identified as a significant pathogen in swine due to its capacity to induce severe intestinal disease and high mortality rates in piglets, which is hitting the pig industry hard. The virus was first identified in 1946 [2] and has been reported in several countries in Africa, Asia, Europe, and America [3,4].

The viral genome length is approximately 28 kb and the 5′ two-thirds contain two open reading frames (ORFs), which encoded polyprotein 1a (pp1a) and 1b (pp1b), respectively [5]. The remaining portion of the genome is primarily responsible for encoding four distinct structural proteins, known as glycoprotein (S), membrane protein (M), nucleocapsid protein (N), and envelope protein (E) [5]. Similar to other coronaviruses, the S protein of TGEV is a type Ⅰ transmembrane glycoprotein that forms homotrimers on the viral membrane surface [6], which is functionally divided into an ectodomain, a transmembrane domain (TM), and a cytoplasmic tail region (CP). Among the major components of the ectodomain are the S1 and S2 subunits. These are responsible for functions such as host receptor binding and fusion with the cellular membrane, respectively [7,8]. Porcine aminopeptidase N (pAPN) or sialic acid were recognized as potential binding receptors to facilitate virion entry into host cells [8,9,10].

CoV infections induce a range of host humoral and cellular immune responses, including the induction of specific neutralizing antibodies and cytotoxic T lymphocyte responses, which collectively aim to eliminate viral infection [11]. S protein is a crucial target antigen for neutralizing antibodies against virus infection. It is composed of multi-epitopes that have immunoprotective effects and elicit neutralizing antibodies and the cytotoxic cellular response, making it an ideal antigenic prime component. Currently, there is a paucity of knowledge regarding its antigenic and immunogenic profiles. Computer-based immunoinformatic approaches are a unique technique for designing effective epitope-based vaccines with a lot of success that have advantages over traditional vaccine development techniques, such as improved stability and specificity as well as decreased time and cost [12,13]. Thus, combining computer-based immunoinformatic and molecular dynamics offers a great strategy to develop an effective subunit vaccine for combating this infectious disease. Currently, numerous research groups are creating chimeric vaccines against a range of pathogens. The approach has been employed to target a number of diseases, including leishmaniasis, dengue fever, onchocerciasis, and related filarial disease, as well as SARS-CoV-2 and cancer [14,15]. Several multi-epitope vaccine candidates have demonstrated considerable promise, such as EMD640744, undergoing a phase 1 clinical trial, which is a notable example [16]. Cheng et al. designed a novel epitope-based vaccine against tuberculosis infection based on immunoinformatic predictions, and the results reveal that this vaccine can effectively elicit immune responses in in silico analysis and animal experiments [17]. Eickhoff et al. used immunoinformatic tools to identify highly conserved T cell epitopes in diverse influenza A strains that strongly support universal multi-epitope T cell-targeting influenza A vaccines [18]. Collectively, computational vaccinology has enormous potential to facilitate epitope-based vaccine development.

Traditional vaccines contain the whole organism or several components, which not only wastes immunization resources but also has the potential to trigger allergic reactions. In contrast, epitope-based vaccines are made up of non-toxic and highly antigenic epitope fragments that can induce strong immune responses, which can reduce allergic reactions. However, screening effective antigenic epitopes is a major challenge since they usually contain a limited number of amino acids and are weakly immunogenic. At the same time, there have been few or no studies applying immunoinformatics to the development of multi-epitope TGEV vaccines. Therefore, in this study, we employed immunoinformatic approaches together with molecular docking molecular dynamic analysis and immune simulation to investigate the potential immunogenic profiles of S protein and construct a multi-epitope vaccine. The immunoinformatic approaches presented here offer a novel perspective on the engineering of a subunit vaccine, particularly regarding epitope-based vaccine design and serological diagnosis, supporting the prevention of TGEV infection.

## 2. Results

### 2.1. Conservative Analysis of TGEV S Protein and Selection of Vaccine Strain

**Figure 1:**
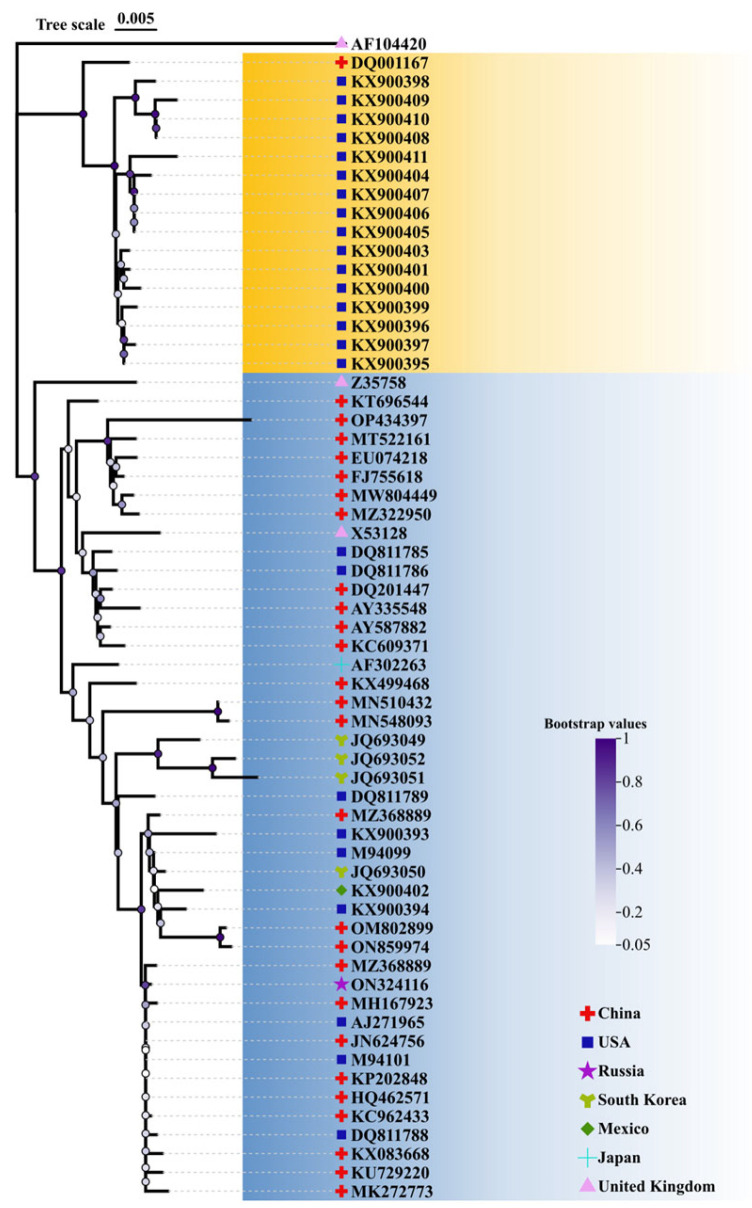
Phylogenetic tree of the TGEV S protein. Neighbor-joining (NJ) tree was reconstructed based on S protein amino acid sequences using the p-distance model with 1000 bootstrap replicates.

To select a representative strain for vaccine design, 62 TGEV S protein sequences were retrieved from GenBank and subsequently analyzed using MEGA-X (Table S1). The high frequency of mutations of S protein amino acid residues is shown in Table S2, and 14 of 25 mutations were present in the S1 subunit. Of note, 7 of 25 mutations occurred in the D∅ domain, implying that the D∅ domain is highly varied among these 62 S protein sequences. Finally, the conserved TGEV S protein (AKA60054) was selected as a representative strain for further epitope prediction (Figure 1).

### 2.2. S Protein Structure Simulation and Glycosylation Site Prediction

**Figure 2:**
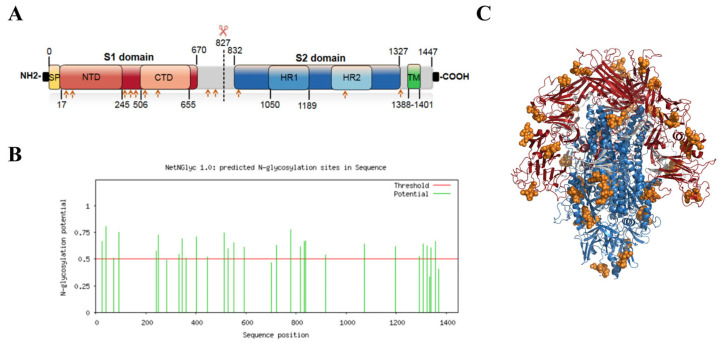
Analysis of N-glycosylation sites of TGEV S protein. (**A**) Schematic diagram of TGEV S protein. SP: signal peptide; NTD: N-terminal domain; CTD: C-terminal domain; HR1: heptad repeat region 1; HR2: heptad repeat region 2; TM: transmembrane. Predicted N-glycosylation sites are marked with orange arrows. (**B**) NetNGlyc 1.0 server results for TGEV S protein (AKA60054). (**C**) S1 subunits are indicated in red and S2 subunits in blue. N-glycosylation sites are shown as orange spheres in the TGEV S protein trimer.

TMHMM-2.0 showed that residues 1 to 1387 aa were an extracellular domain, and the red line indicated that 1388 to 1410 aa could form a typical transmembrane helix region, whereas 1411 to 1447 aa were located as an intracellular domain (Figure 2A). The NetNGlyc 1.0 server result showed that the TGEV S protein contained 13 potential N-glycosylation sites (Figure 2B), and structural simulations showed that all N-glycosylation sites were on the S-protein trimer surface (Figure 2C).

### 2.3. Prediction and Analysis of B Cell Epitopes

**Figure 3:**
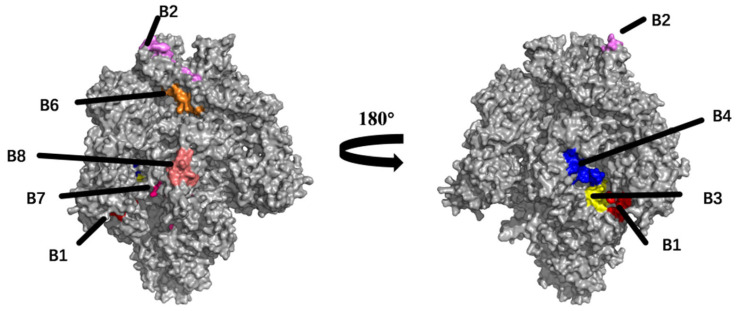
Localization of predicted linear B cell epitopes of the TGEV S protein. The TGEV S protein predicted 8 linear B cell epitopes, which exhibited distinct colors (B1: red; B2: purple; B3: yellow; B4: blue; B6: orange; B7: pink; B8: flesh pink).

The BepiPred 2.0 and ABCpred servers were used to predict eight potential linear B cell epitopes. All the predicted epitopes were highly conserved (>85% identity) and epitope B7 was strictly conserved in 62 TGEV strain sequences (Table 1). These B cell epitopes were displayed on the surface of the S protein trimer, and Figure 3 demonstrates that seven epitopes were forecast in the accessible surface area of the S protein and marked with distinct colors.

### 2.4. T Cell Epitopes Prediction

**Figure 4:**
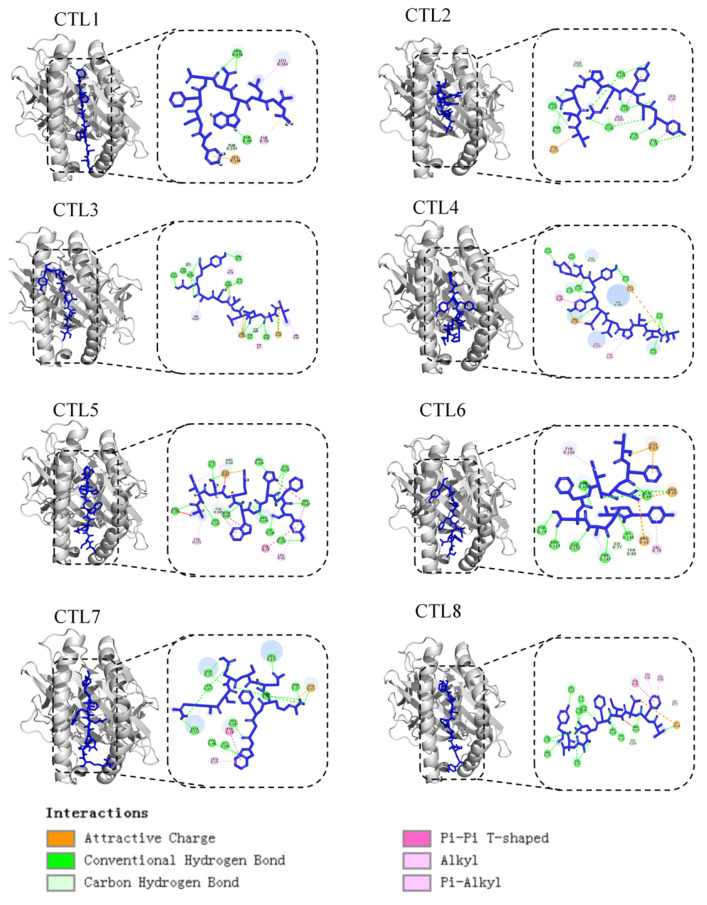
Molecular docking analysis of TGEV peptides (shown in blue) with SLA-1*0401 protein (shown in grey).

In all, eight peptides were predicted as CTL epitopes following analysis of antigenicity using VaxiJen v2.0 and peptide-MHC-Ⅰ binding affinity using the CTLpred server (Table 2). Among these eight CTL epitopes, five shared 100% identity in 62 TGEV strain sequences. The peptides were created using SYBYL-X2.1.18, and each peptide was further docked with SLA-1*04:01 molecules using ClusPro-2.0. Figure 4 shows that all eight peptides docked into the groove of the SLA-1*04:01 molecule and formed stably. The ∆G for each epitope binding reaction ranged from −9.4~−2.6 kcal/mol (Table S3). Th cell epitopes were selected with reference to NetMHCpan 4.0 predictions and confirmed the last six potential Th cell epitopes (Table 3).

### 2.5. Design of Multi-Epitope Vaccine Candidate

**Figure 5:**
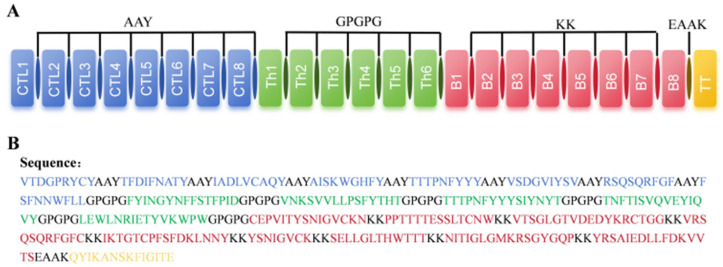
Design and construction of a multi-epitope vaccine. (**A**) Graphical representation of a TGEV multi-epitope vaccine construct. (**B**) Candidate vaccine sequence.

Linker selection is a non-negligible part of the design of multi-epitope vaccines and affects immunogenicity, efficacy, and stability. AAY and GPGPG linkers can enhance antigen recognition and KK linkers can effectively increase the folding and thermal stability of proteins. Thus, to construct a multi-epitope vaccine, these linkers were considered. Additionally, a TT peptide sequence was fused into the C-terminal end of the vaccine via an EAAK linker, which aimed to improve its immunogenicity. Finally, as demonstrated in Figure 5, a multi-epitope vaccine containing 358 amino acids was designed based on immunoinformatics methods.

### 2.6. Antigenicity, Allergenicity, and Physicochemical Properties Evaluation of Vaccine Candidate

**Figure 6:**
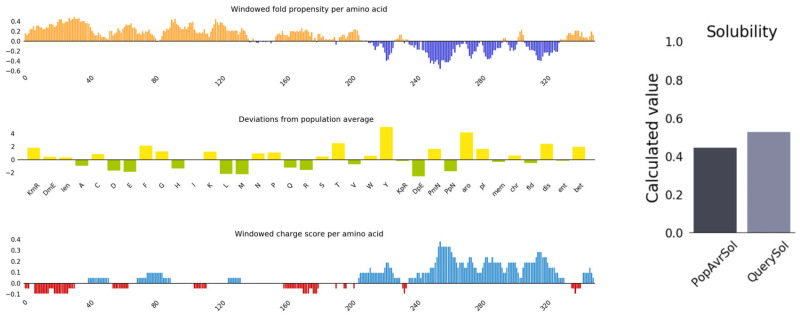
Solubility prediction of the designed vaccine.

The antigenicity analysis revealed that the multi-epitope vaccine had an antigenicity score of 0.49 (>0.4, default threshold), indicating that it was a good vaccine candidate. The physicochemical properties were calculated using the Expasy-ProtParam. The molecular weight was 39.9 kDa and the theoretical isoelectric point (pI) = 9.32. The instability index = 36.14, with a grand average of hydropathicity (GRAVY) = −0.324, indicating that the protein in question was stable. The predicted half-lives in mammalian reticulocytes, *Escherichia*, and yeast were greater than 3, 10, and 20 h, respectively. The AllerTop v.2.0 server was determined to be non-allergenic. The Protein-Sol program predicted a solubility probability = 0.548 in *E. coli* (>0.45, default threshold) (Figure 6). Based on these data, it is suggested that the designed multi-epitope vaccine candidate has good immunogenicity, high thermostability, and a stable expression in *E. coli*.

### 2.7. Secondary and Tertiary Structure Prediction of Vaccine

**Figure 7:**
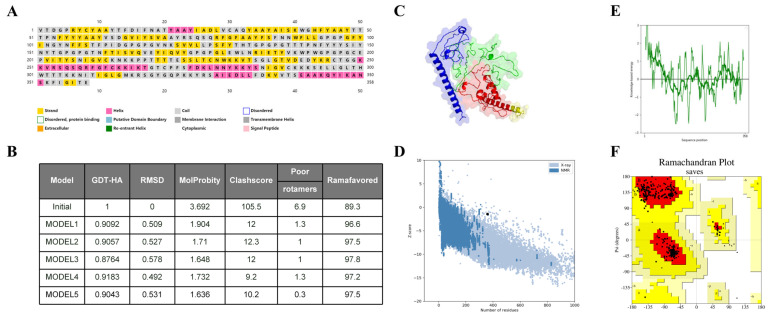
Verification of generated TGEV vaccine tertiary structure. (**A**) PSIPRED server prediction results. (**B**) The model refined by the Galaxy WEB server. (**C**) The tertiary structure of refined modeled TGEV vaccine. The CTL epitope, Th cell epitope, B cell epitope, and TT peptide are shown in blue, green, red, and yellow, respectively. (**D**) The Z-score of TGEV vaccine tertiary structure. (**E**) Plotting the energy. (**F**) Ramachandran plot analysis, where the red areas indicate the most favoured regions.

The secondary structure prediction result of the PSIPRED server revealed that the vaccine candidate had 34.3% alpha helix, 13.6% beta strand, and 51.6% random coil in the structure (Figure 7A). The 3D Pro server performed tertiary structural simulations and generated a total of five candidate models, and the suitable model (MODEL5) was chosen based on quality assessment. The parameters of the model were GDT-HA (0.9043), RMSD (0.531), Mol Probity (1.636), Clash score (10.2), Poor rotamers (0.3), and Rama-favored (97.5) (Figure 7B). Furthermore, the z-value = −1.5 (Figure 7D) and the known-based energy was predominantly lower (Figure 7E). A Ramachandran diagram produced by a PROCHECK server and the refining model revealed that residues with 92.9%, 7.1%, and 0.0% were the most favored, additionally allowed, and generously allowed regions, respectively (Figure 7F). These results demonstrated that this tertiary structure is sufficient to support subsequent exploration.

### 2.8. Prediction of Conformational B Cell Epitopes

**Figure 8:**
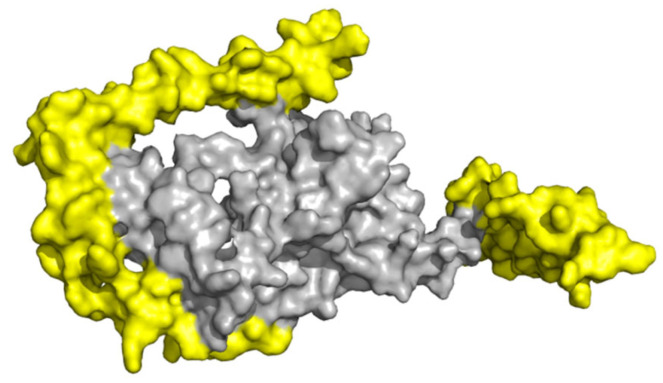
Prediction of conformational B cell epitopes. The predicted conformational B cell epitopes are shown in yellow.

Ellipro server predictions showed four potential conformational B cell epitopes in the previously modeled tertiary structures. Their scores were in the range of 0.658 to 0.819 (threshold > 0.6) (Table S4). Figure 8 highlights the epitopes of the tertiary structures.

### 2.9. Molecular Docking with TLR4

**Figure 9:**
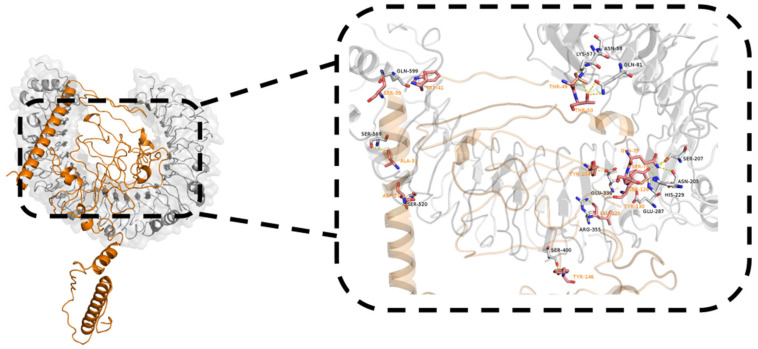
Molecular docking result of designed TGEV vaccine and TLR4 via HDOCK server. TGEV vaccine–TLR4 complex is shown as sticks. TGEV vaccine and TLR4 are shown in orange and grey, respectively. Amino acid residues with interacting weights are shown in stick, with oxygen atoms represented in red and nitrogen atoms in blue.

TLRs have been identified as a kind of protein molecule that can stimulate innate immunity. In particular, TLR4 can elicit the production of inflammatory cytokines via the recognition of viral structural glycoproteins [19,20]. Thus, the structural interaction between the vaccine candidate and TLR4 was investigated via the HDOCK server. The results showed that there are 10 docking complexes and the lowest global energy was selected (Table S5), with the KD value and ΔG energy being 1.8 × 10^−13^ M and −17.4 kcal/mol at 25.0 °C, respectively (Table S6). The tertiary structure of interacted residues of the docking complex was analyzed with PyMOL-2.6 and showed a complex interaction through 19 hydrogen bonds within 4.0 Å (Figure 9). These results demonstrate that the designed candidate exhibits a robust binding efficiency with TLR4 and can be recognized by TLR4 spontaneously.

### 2.10. Molecular Dynamic Simulation

**Figure 10:**
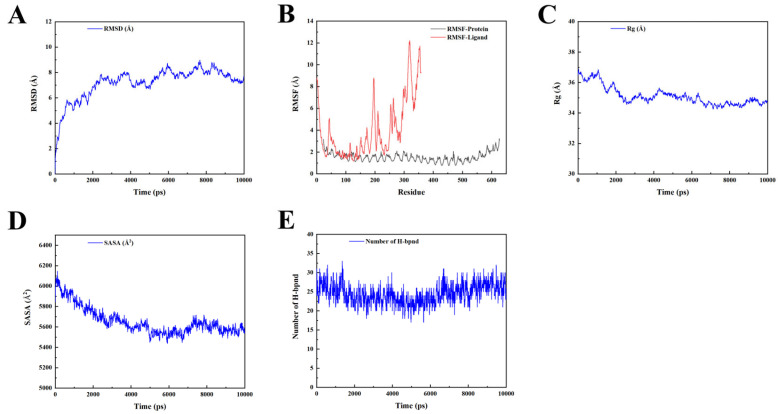
MD simulation results of docked vaccine–TLR4 complex. (**A**) RMSD analysis of the complex. (**B**) RMSF profile of docked complexes. (**C**) Radius of gyration (Rg) analysis of the complex. (**D**) Solvent accessible surface area (SASA) analysis of the complex. (**E**) Hydrogen bonds analysis of the complex.

The research in this subsection was realized through GROMACS-2019. The RMSD value of the complex was stable at 7 Å (Figure 10A), as the RMSD of the ligand was considered to be stable within 10 Å. Furthermore, the RMSF result shows that regions 200–250 and 300–350 aa of the complex demonstrated significant fluctuation (Figure 10B). Figure 10C shows consistent behavior in terms of the radius of gyration (Rg) value, which remained at around 27 Å, with no significant deviation during the simulation period. As shown in Figure 10D, the decrease in solvent-accessible surface area (SASA) from 6000 to 5600 A^2^ supports the possibility that the surface area of the protein complex was reduced due to vaccine–TLR4 interactions. Hydrogen bond formation was also evaluated throughout the entire simulation, as depicted in Figure 10E; the result reveals that the complex exhibited a robust hydrogen bonding network, indicating stable folding with minimal rearrangement of bonds during the simulation.

### 2.11. Simulation of Immune Responses

**Figure 11:**
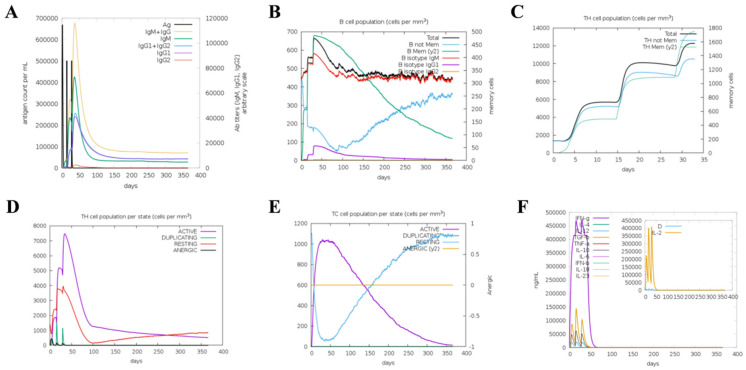
Immune stimulation after TGEV vaccine injection via C-ImmSim server analysis. (**A**) Levels of antibodies. (**B**) The total B cell population. The total Th cell (**C**) and active Th cell (**D**) levels after vaccination. (**E**) The active TC cell populations were stimulated after each injection. (**F**) The concentration of cytokines.

The potential of vaccines to elicit humoral and cellular immunity is essential for combating viruses. To evaluate the immunological efficacy, we used the C-ImmSim server to assess the immune responses induced by this designed vaccine candidate. Figure 11A reveals that the vaccine can induce three peaks in antibody levels following each injection. After the primary injection, IgM+IgG, IgM, and IgG1 antibody levels were slightly increased. After the second immunization, the population of IgM+IgG, IgM, IgG1+IgG2, and IgG1 significantly soared. Specifically, the combined IgM+IgG antibody titers reached 650,000 to 680,000/mL. The IgM antibody titer was 450,000/mL, while the titer of combined IgG1+IgG2 reached 220,000/mL after the third injection. Figure 11B shows that the total B cell population was significantly increased after vaccination. The total Th cell and active Th cell populations gradually increased after each injection (Figure 11C,D). Moreover, the active TC cell populations were highly stimulated after each injection (Figure 11E). The interferon γ (IFN-γ) levels significantly increased and reached approximately 450,000/mL. Similarly, the levels of IL-2 reached approximately 150,000/mL (Figure 11F). Ultimately, these results demonstrate that the developed vaccine is effective in inducing an organismal immune response after three immunizations in mammals and has a protective effect on animals. Unfortunately, the limited capacity of the C-ImmSim server did not allow for the prediction of protective effects after the challenge experiment taking into account the species-specificity of certain viruses, and further data are needed for experimental validation.

### 2.12. Optimization of Codons and In Silico Cloning

**Figure 12:**
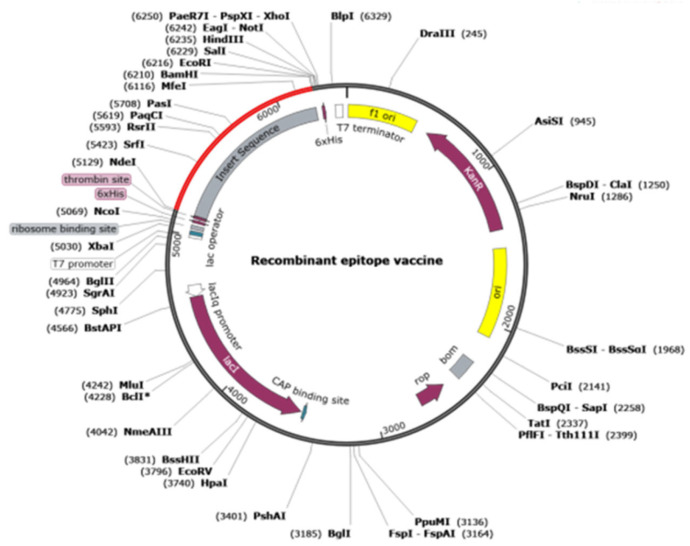
Plasmid mapping of vaccine candidates. The sequence was cloned into a pET28a (+) vector, shown in red.

The preparation of proteins using *E. coli* has the advantages of low cost, easy operation, and high expression. Therefore, we optimized the codon using the JCAT tool. The final optimized results showed an adaptation index (CAI) value = 1 and GC contents = 50%. In general, a CAI value higher than 0.8 and optimal GC contents ranging from 40 to 70% are considered to indicate stable expression. We selected the pET-28a (+) prokaryotic expression vector using *XbaI* and *ECoRI* restriction endonuclease sites for cloning the vaccine gene sequence by SnapGene, as depicted in Figure 12.

## 3. Discussion

CoVs are highly contagious zoonotic potential pathogens with hosts such as bats, pangolins, humans, pigs, dogs, cats, mice, poultry, and so on [21]. The emergence of COVID-19 poses a major threat to the safety of the world’s population, once again drawing attention to coronaviruses. The same as the porcine enteric CoV, TGEV was responsible for a severe threat to pig farms and became a challenge to the prevention of swine diseases. Investigators have been trying to explore TGEV to comprehend its structural biology, evolution dynamics, preventive measures, and other pertinent aspects [22,23].

Up to now, vaccination has been one of the most effective measures to control this infectious disease, and a variety of traditional inactivated or attenuated vaccines based on the whole virus have been commercialized [22]. However, the novel emerging TGEV strains have brought severe challenges to current commercial vaccine applications. Thus, there is an obvious need to develop an efficacious vaccine that will provide promising protection against the diversity of novel variants.

Currently, conventional TGEV vaccines play an effective role in combating TGEV infection in the clinic. What can not be ignored is that the use of live-attenuated TGEV vaccines may potentially elevate the risk of reversion to virulence, which could ultimately lead to the emergence of novel variants. In contrast, inactivated vaccines are generally considered safer than live-attenuated vaccines owing to infectious nucleic acid, whereas their weak ability to elicit T cell immune responses makes them less protective. Importantly, CTL-mediated immune responses are critical for eliminating virus-infected cells. Thus, an ideal TGEV vaccine should stimulate an effective T cell response. To date, there are still many blank areas in research for TGEV subunit vaccines, such as a lack of randomized clinical trials or research projects that have received national approval. Computer-based immunoinformatic approaches can be applied for predicting and analyzing viral antigens and potential epitopes and evaluating antigenic immunogenicity that induces strong immune responses; in addition, they may decrease the time and cost needed for developing novel vaccines against emerging variants, which is of great benefit to designing epitope-based subunit vaccines [24]. Furthermore, only epitopes (B and T cell) that are highly conserved, immunogenic, and antigenic and that, at the same time, can be effectively recognized, are selected for the purpose of effectively stimulating a strong immune response. Importantly, the side effects of non-essential epitopes are minimized, which may solve the limitations of traditional vaccines [24]. To sum up, multi-epitope-based subunit vaccines are an attractive option to traditional inactivated and live-attenuated vaccines due to their high level of safety and ability to elicit a specific immune response to corresponding epitopes.

In this study, we developed an ideal multi-epitope vaccine that targets TGEV S proteins to stimulate strong humoral and cellular immune responses. In concrete terms, we attempted to deepen our molecular and immunological understanding of the TGEV S protein and vaccine design principles by employing various accurate and advanced tools. To improve the accuracy of predicting B cell epitopes, we used two tools (BepiPred-2.0 and ABCpred) for epitope prediction simultaneously. After filtering, eight conserved linear B cell epitopes were screened in the S protein, and seven of the eight epitopes were located on the surface of the S protein trimer. In addition, an extensive glycan shield decorated the S protein, thereby blocking neutralizing antibody recognition. It was reported that the SARS-CoV-2 S protein utilizes a glycan shield to thwart the host immune response [25]. This is a factor that researchers must consider when developing a subunit vaccine based on a highly glycosylated protein. Therefore, none of the B cell epitopes that contained N-glycosylation sites was selected in this study. According to these results, we found that three selected B cell epitopes (B3, B4, and B8) are located in a glycan hole that could be exploited for epitope-focused antigen design, enhancing antibody recognition [26]. Furthermore, epitope B7 (YSNIGVCK) was strictly conserved in all the TGEV strains; notably, this epitope has been identified as a neutralizing epitope region in the S protein of porcine epidemic diarrhea virus (PEDV) [27], indicating a greatly promising epitope that could be used for a bivalent subunit vaccine against TGEV and PEDV.

Apart from antibody-mediated immunity, CTL and Th cell-mediated cellular immunity are crucial elements in eliminating viruses. To further screen CTL epitopes, we predicted eight potential CTL epitopes derived from the S protein, docked these peptides with SLA-1*04:01, and demonstrated that all eight peptides formed stable hydrogen bonds with the residues of the SLA, with low ΔG energies (−9.4 to −2.6 kcal/mol). The results indicate that they have a high binding affinity with the SLA molecule, supporting the accuracy of predicting CTL epitopes. Importantly, four CTL epitopes (CTL1, CTL5, CTL6, and CTL7) were strictly conserved among 62 isolates with high immunogenicity scores (0.9093, 0.7548, 1.1525, and 0.608) and may provide wide protection against various TGEV strains, which should be experimentally investigated in further studies.

The selection of the linker is of paramount importance in the development of recombinant fusion proteins as the appropriate linker can improve biological activity, increase expression yield, and avoid unnecessary toxic epitopes [28]. AllerTop v.2.0 showed that the vaccine candidate is non-allergenic and the VaxiJen tool revealed its high immunogenicity. Generally, the antigen epitopes presented on MHC-II could activate Th1 and Th2 cells, consequently, and release specific subtypes of cytokines. Th1 cells can produce IFN-γ and eliminate the cellular virus, whereas Th2 cells mainly produce IL-4, which could activate the proliferation and differentiation of antigen-presenting cells (APCs) [29]. The C-ImmSim server results revealed that the vaccine can induce robust humoral and cellular immune responses and the highest level of IFN-γ production, with a high level of IL-2. Additionally, there was a low level of IL-4 and IL-10, indicating that the vaccine favors Th1-biased cell-mediated immune responses. Immunoinformatics analyses predicted potentially strong epitopes (B and T cells) that were not only used in this experiment to design a vaccine in tandem but could also help early stages of the development of other vaccines or drugs to combat TGEV infection. This study was pioneering and exploratory, and, even though there are great benefits, there were some drawbacks due to external conditions. First, the MHC-II binding CD4 T cell epitopes of the candidate vaccine were originally designed to bind the human HLA-II allele rather than the SLA allele, which may lead to a decrease in recognition efficiency for the SLA allele. Although it has been suggested that HLA-II can be utilized to make predictions for other mammals, the accuracy of such predictions may vary [30]. Secondly, its prediction accuracy can be affected by the accuracy of the algorithm and the amount of database information used by the prediction tool, etc. Finally, despite the numerous benefits associated with this computational study, these predicted epitopes should be experimentally verified.

## 4. Materials and Methods

**Figure 13:**
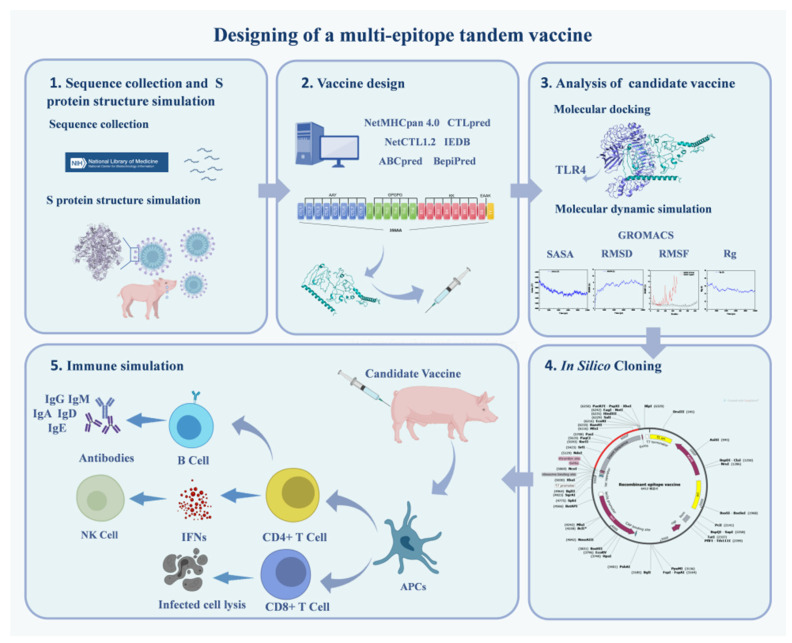
Schematic of illustration-specific design process using immunoinformatic approaches.

Figure 13 illustrates the entire methodology of this study.

### 4.1. Sequence Datasets and Phylogenetic Tree Analysis

The TGEV S information from 7 countries was obtained (summarized in Table S1). In total, 62 strain sequences were available from the National Center for Biotechnology Information (NCBI) database (https://www.ncbi.nlm.nih.gov). MEGA-X software was used to align the sequences and reconstruct a phylogenetic tree. Furthermore, to gain a deeper understanding of amino acid mutation patterns in the S protein, high-frequency mutations were analyzed using the BV-BRC on 1 March 2024.

### 4.2. Tertiary Structure Simulation and N-Glycosylation Site Prediction of TGEV S Protein

SWISS-MODEL (http://swissmodel.expasy.org) is a commonly used tool for the homology modeling of 3D protein structures [31]. The tertiary structure of the TGEV S protein was reconstructed from the PEDV S protein structure (PDB ID: 6u7k) using the SWISS-MODEL server. The N-glycosylation sites were predicted from the sequence information by NetNGlyc-1.0 on 1 March 2024. (https://services.healthtech.dtu.dk/services/NetNGlyc-1.0/).

### 4.3. Prediction of B Cell Epitopes

BepiPred 2.0 was used to predict linear B cell epitopes on 1 March 2024 [32], with a cut-off of 0.5, and epitopes of more than 8 amino acid residues were considered for further analysis. To predict the epitopes more accurately, ABCpred’s forecast results were also considered [33].

### 4.4. T Cell Epitope Prediction

For the adaptive immune response, it is critical that MHC-I antigen peptides meet the following requirements: exposure to target cells and recognition by cytotoxic CTL. IEDB MHC-I and CTLpred were used to predict CTL epitopes. For IEDB MHC-I prediction, peptides with a percentile score < 10 were considered high-affinity binders, and, for CTLpred, we used an SVM-based module with default parameters. The accuracy of the QM (70.0%), ANN (72.2%), and SVM (75.2%) methods varied slightly [34]. An artificial neural network-based NetMHCIIpan 4.0 was used to make predictions of CD4 T cell epitopes. The IFN-γ and IL-4 inducibility of the epitopes was predicted via the IFN epitope server and IL-4 pred server, respectively (1 April 2024) [29,35].

### 4.5. Molecular Docking of CD8 T Cell Epitopes with SLA Alleles

The unwanted peptides and water molecules in the complex (PDB ID: 3QQ3) were deleted by PyMOL-2.6 to obtain a usable SLA-1*0401. Before docking, CD8 T cell epitopes were created using SYBYL-X2.1.1. The molecular docking was carried out in ClusPro 2.0 software (10 April 2024) [36]. Finally, the molecular interactions were visualized via Discovery Studio 2022.

### 4.6. Antigenicity, Allergenicity, Toxicity, and Conservation Analysis

All epitopes were analyzed using AllerTOP-2.0 (https://www.ddg-pharmfac.net/AllerTOP/method.html) and ToxiPred-2.0 (https://webs.iiitd.edu.in/raghava/toxinpred2/) to assess their potential allergenicity and toxicity, respectively (1 April 2024) [37,38]. AllerTOP v.2.0 identifies allergens using the k nearest neighbor (kNN) approach and the accuracy rate is about 85%. The web server ToxinPred was developed on the foundation of a machine learning technique and a quantitative matrix, which were employed to take advantage of the distinctive properties of peptides [38]. The VaxiJen 2.0 server (https://www.ddg-pharmfac.net/vaxijen/VaxiJen/VaxiJen.html) scores antigens based on amino acid sequences, with default scores greater than 0.4 considered to be viral antigens (1 April 2024) [39]. The predicted epitopes were aligned with 62 isolates using the IEDB-conservancy tool, with sequence identity greater than 85% as the standard [40].

### 4.7. Construction of the Vaccine, Structure Modelling, and Validation

The AAY, GPGPG, and KK linker were used to connect CTL, TH, and B cell epitopes, respectively. Then, the TT-peptide was fused at the C-terminus via EAAK to obtain the complete sequence. ExPASy (https://www.expasy.org/) was used to predict the physiochemical properties (10 April 2024) [41]. Protein-sol (https://protein-sol.manchester.ac.uk/) was used to predict the solubility of the final designed fusion protein (10 April 2024).

### 4.8. Secondary and Tertiary Structure Prediction

The construct’s secondary structure was predicted by the PSIPRED4.0 server (http://bioinf.cs.ucl.ac.uk/psipred) (25 April 2024) [42]. The SCRATCH: A Quick Description server (http://scratch.proteomics.ics.uci.edu/index.html) was used to generate the tertiary structure (25 April 2024). The galaxy server was used for loop remodeling and structure refinement of the 3D structure [43]. Additionally, the validity and rationality of the tertiary structure were verified by using different online servers. Specifically, the ProSA-web server (https://prosa.services.came.sbg.ac.at/prosa.php) was used to evaluate the quality of the modeled tertiary structure (25 April 2024). Ramachandran analysis was achieved by Molprobity (http://molprobity.biochem.duke.edu/) (25 April 2024).

### 4.9. Prediction of Conformational B Cell Epitopes

To validate the elicitation of B cell response, the IEDB-Ellipro server (http://tools.iedb.org/ellipro/) was used to predict the conformational B cell epitopes.

### 4.10. Molecular Docking of Vaccine with TLR4 and Molecular Dynamic Simulation

Toll-like receptor 4 (TLR4), a pivotal molecule in pathogen recognition and immune response, serves as a receptor for coronavirus S protein recognition [44]. Blind docking for TLR4 (PDB ID:4G8A) and the candidate vaccine was performed using the HDOCK server [45]. Furthermore, the ΔG of the top 10 results was predicted by PDBePISA online on 29 April 2024 (https://www.ebi.ac.uk/pdbe/pisa/). To evaluate the dynamic changes of the designed vaccine with the receptors, the molecular dynamic (MD) simulation of the suitable model was performed by using GROMACS-2019.6 [46]. AMBER99SB-ILDN was employed for the generation of protein topologies. We chose the appropriate size of the simulation box to prevent atoms from overflowing during the simulation. In general, greater than 1.0 nm was a safe distance between the atom and the box. The aforementioned system was rendered electrically neutral by the introduction of SPC216 water molecules, Na+, and Cl− into the box. Subsequently, the MD simulation was performed for 10 ns at 300 K and 1 bar [47].

### 4.11. Simulation of Immune Responses against Designed Vaccines

To determine the immune response of the vaccine, the C-Immune Simulation (https://kraken.iac.rm.cnr.it/C-IMMSIM/) was used to investigate in silico immune profiles (29 April 2024). In total, 3 injections were conducted at 0, 28, and 42 days, and all the input parameters were set as default, with 1000 simulation steps.

### 4.12. Codon Optimization and In Silico Cloning

To obtain superior yield of a recombinant protein in *E. coli*, the JCat Tool was used for codon adaptation (1 May 2024) [48]; it is recommended that the GC content range is 40–70%. Then, the design was cloned into a pET-28a (+) using *XbaI* and *EcoRI*.

## 5. Conclusions

In the present study, we predicted and screened potential B and T cell epitopes in TGEV S proteins based on immunoinformatics and selected appropriate linkers. The objective was to create a multi-epitope vaccine. To validate the actual efficacy of the designed object as far as we could, the interaction of the vaccine candidate with TRL4 was simulated by molecular dynamics, and in vivo immunological simulation was also effective in stimulating high titers of antibodies (IgG and IgM) and antiviral correlation cytokines (IFN-γ and IL-2). Importantly, the vaccine favors Th1-biased cell-mediated immune responses. E. coli was chosen to prepare the vaccine candidate, taking into account both the economic cost and the ease of operation. This study provided a promising approach to design a subunit vaccine against TGEV that is non-toxic, highly immunogenic, stable, and cost-effective. Furthermore, the approach demonstrates the development of prevention strategies against TGEV and has great potential to inform significant implications for combating other CoVs.

## Figures and Tables

**Table 1:** Predicted linear B cell epitopes.

Epitope	Length	Position	Sequence	Toxin	Identity (62 Isolates)
B1	14	137–150	PPTTTTESSLTCNW	Non-Toxin	87.09% (55/62)
B2	16	530–545	NITIGLGMKRSGYGQP	Non-Toxin	96.77% (60/62)
B3	12	955–970	YRSAIEDLLFDKVVTS	Non-Toxin	93.54% (58/62)
B4	18	968–985	VTSGLGTVDEDYKRCTGG	Non-Toxin	91.93% (57/62)
B5	11	1186–1196	VRSQSQRFGFC	Non-Toxin	96.77% (60/62)
B6	16	603–618	IKTGTCPFSFDKLNNY	Non-Toxin	85.48% (53/62)
B7	8	802–809	YSNIGVCK	Non-Toxin	100.00% (62/62)
B8	12	757–768	SELLGLTHWTTT	Non-Toxin	98.38% (61/62)

**Table 2:** Predicted CTL epitopes of the TGEV S protein.

Epitope	Length	Position	Sequence	Score	Rank	TAP Score	Proteasome Score	MHC Ⅰ IC_50_ (nM)	Toxin	Identity (62 Isolates)
CTL1	9	275–283	FSFNNWFLL	0.90876	0.15	0.43	1.79	307.9	Non-Toxin	100.00% (62/62)
CTL2	9	391–399	VTDGPRYCY	0.968509	0.02	1.2	2.75	62.2	Non-Toxin	93.54% (58/62)
CTL3	9	421–429	AISKWGHFY	0.871218	0.07	1.39	2.68	42.3	Non-Toxin	96.77% (60/62)
CTL4	9	724–732	VSDGVIYSV	0.776052	0.23	1.25	1.32	210.6	Non-Toxin	100.00% (62/62)
CTL5	9	766–774	TTTPNFYYY	0.745044	0.17	1.21	1.54	67.7	Non-Toxin	100.00% (62/62)
CTL6	9	988–996	IADLVCAQY	0.880338	0.06	1.17	1.46	90.8	Non-Toxin	100.00% (62/62)
CTL7	9	1187–1195	RSQSQRFGF	0.418621	0.04	1.15	1.22	148.8	Non-Toxin	100.00% (62/62)
CTL8	9	1329–1337	TFDIFNATY	0.818436	0.12	1.35	1.28	98	Non-Toxin	96.77% (60/62)

Note: Smaller IC_50_ values indicate higher affinity. IC_50_ values = 50 and 500 nM were considered to be the cut-off between high and intermediate affinity and affinities greater than 500 nM were not considered.

**Table 3:** Predicted Th cell epitopes of the TGEV S protein.

Epitope	Length	Position	Sequence	Rank	IL-4	IFN	MHC Ⅱ IC_50_ (nM)	Toxin	Identity (62 Isolates)
Th1	15	428–442	FYINGYNFFSTFPID	0.84	IL4-inducer	Positive	45.63	Non-Toxin	91.93% (57/62)
Th2	15	513–527	VNKSVVLLPSFYTHT	0.37	IL4-inducer	Positive	32.82	Non-Toxin	85.48% (53/62)
Th3	15	766–780	TTTPNFYYYSIYNYT	2.9	IL4-inducer	Positive	160.09	Non-Toxin	100.00% (62/62)
Th4	15	796–810	CEPVITYSNIGVCKN	1.1	IL4-inducer	Positive	31.1	Non-Toxin	100.00% (62/62)
Th5	15	837–851	TNFTISVQVEYIQVY	3.9	IL4-inducer	Positive	192.33	Non-Toxin	98.38% (61/62)
Th6	15	1375–1389	LEWLNRIETYVKWPW	5	IL4-inducer	Positive	63.01	Non-Toxin	100.00% (62/62)

## Data Availability

Data are contained within the article and Supplementary Materials.

## Supplementary Materials

The following supporting information can be downloaded at: https://www.mdpi.com/article/10.3390/ijms25168828/s1.
