# Supplementary material for: Designing a Candidate Multi-Epitope Vaccine against Transmissible Gastroenteritis Virus Based on Immunoinformatic and Molecular Dynamics
Source: Int J Mol Sci. 2024 Aug 13;25(16):8828. doi: 10.3390/ijms25168828 (PMC11354480; doi:10.3390/ijms25168828)
Supplement: Supplementary file 1 [file ijms-25-08828-s001.zip › ijms-3104444-supplementary.pdf]

Supplementary Table S1 Information of TGEV strains in this study

GenBank accession no.	Strain name	Country	Collection date
AJ271965	PUR46-MAD	USA	1964
DQ811785	Miller M6	USA	1965
DQ811786	partially attenuated Miller M60	USA	1987
DQ811788	attenuated Purdue P115	USA	Not Available
DQ811789	virulent Purdue	USA	1952
KX900393	TGEV/USA/Z/1986	USA	1986
KX900394	TGEV/USA/HB/1988	USA	1988
KX900395	TGEV/USA/Minnesota138/2006	USA	2006
KX900396	TGEV/USA/Illinois139/2006	USA	2006
KX900397	TGEV/USA/NorthCarolina140/2007	USA	2007
KX900398	TGEV/USA/Minnesota141/2007	USA	2007
KX900399	TGEV/USA/NorthCarolina142/2007	USA	2007
KX900400	TGEV/USA/Iowa143/2008	USA	2008
KX900401	TGEV/USA/Tennessee144/2008	USA	2008
KX900403	TGEV/USA/Illinois146/2008	USA	2008
KX900404	TGEV/USA/Oklahoma147/2012	USA	2012
KX900405	TGEV/USA/Minnesota148/2013	USA	2013
KX900406	TGEV/USA/Illinois149/2013	USA	2013
KX900407	TGEV/USA/Minnesota150/2013	USA	2013
KX900408	TGEV/USA/Wisconsin151/2014	USA	2014
KX900409	TGEV/USA/Minnesota152/2014	USA	2014
KX900410	TGEV/USA/Minnesota153/2014	USA	2014
KX900411	TGEV/USA/SouthDakota154/2014	USA	2014
M94099	NEB72-RT	USA	1972
M94101	PUR46-MAD	USA	Not Available
MH167923	HQ2016	China	2016
MK272773	FS-WS	China	2017
MN548093	TGEV/CH/HB/FP/2017	China	2017
MT522161	HLJ-17	China	2020
MW804449	CH8438	China	2017
MZ322950	CH/GX/TGEV/2662/2019	China	2019
MZ368889	HB-1	China	2020

OM802899	SZ19	China	2019
ON016092	CHN-SC-H	China	Not Available
ON859974	HNSQ-2021	China	2021
OP434397	HN-2012	China	2012
Z35758	TFI	China	1983
EU074218	attenuated H165	China	Not Available
FJ755618	H16	China	1973
HM776941	AYU	China	2009
HQ462571	WH-1	China	2010
JN624756	HR/DN1	China	2010
KC609371	ZH	China	Not Available
KC962433	TGEV-HX	China	2012
KP202848	SHXB	China	2013
KT696544	JS2012	China	2012
KU729220	TH-98	China	1998
KX083668	HE-1	China	2015
KX499468	TGEV AHHF	China	2015
AY335548	TS/2003	China	2003
AY587882	HN2002	China	2002
DQ001167	TSX	China	2005
DQ201447	TS/2005	China	2005
JQ693049	133	South Korea	1997
JQ693050	DAE	South Korea	2012
JQ693051	KT2	South Korea	2000
JQ693052	KT3	South Korea	2000
AF104420	96-1933	United Kingdom	1996
X53128	TGEV FS772/70	United Kingdom	1990
AF302263	TO14	Japan	2000
KX900402	TGEV/Mex/145/2008	Mexico	2008
ON324116	Vaccine 462	Russia	1972

Supplementary Table S2 High frequency mutations of S protein amino acid residues in 63 TGEV strains

Subunit	Domian	Position	Consensus	Alignment detail
S1	NTD	31	Asn	Asn=45,Lys=17,Thr=1
		32	His	Arg=1,Gln=24,His=37,Tyr=1
		72	Asp	Asn=29,Asp=34
		97	Trp	Ala=1,Arg=1,Asp=1,Leu=4,Ser=10,Trp=46
		184	Ala	Ala=35,Glu=28
		218	Thr	Ala=1,Asn=1,Thr=33,Val=28
		375	Asn	Asn=35,Del=23,Lys=4,Tyr=1
		382	Ser	Phe=29,Ser=33,Tyr=1
		401	Leu	His=24,Leu=39
		416	Ser	Ile=6,Ser=49,Val=8
		485	Tyr	His=25,Tyr=38
	CTD	560	Xaa	Asn=31,Asp=7,His=24,Lys=1
		588	Val	Ile=30,Val=33
		647	Asp	Asp=35,Glu=28
		673	Leu	Leu=37,Val=26
		700	Gln	Arg=2,Gln=49,Lys=12
		784	Thr	Ala=1,Ile=19,Thr=43
		813	Leu	Leu=38,Phe=24,Trp=1
S2		930	Glu	=1,Asp=18,Glu=44
		949	Asp	Asp=36,His=26,Tyr=1
		965	Asp	Ala=11,Asp=32,Ser=20
	HR1	1107	Xaa	Ala=6,Ile=26,Thr=31
		1232	Ala	Ala=37,Gln=1,Pro=21,Ser=3,Thr=1
		1237	Leu	Leu=36,Ser=27
		1342	Gly	Ala=3,Gly=45,Val=15

Supplementary Table S3 Molecular docking result of peptides with SLA-1*0401 protein

Epitope	Hydrogen Bond	Electrostatic	Hydrophobic	ΔG (kcal/mol)
CTL1	6	1	3	-9.4
CTL2	16	1	7	-5.3
CTL3	16	1	9	-5
CTL4	21	2	8	-7
CTL5	22	1	11	-4.5
CTL6	15	3	3	-6
CTL7	17	1	3	-2.6
CTL8	21	1	4	-6.7

Supplementary Table S4 The conformational B cell epitopes of the TGEV vaccine predicted by the ElliPro

No.	Start	End	Peptide	Length	Score
1	298	358	LTHWTTTKKNITIGLGMKRSGYGQPKKYRSAIEDLLFDKVVTSEAAKQYIKANSKFIGITE	61	0.819
2	187	201	TYVKWPWGPGPGCEP	15	0.772
3	1	79	VTDGPRYCYAAYTFDIFNATYAAYIADLVCAQYAAYAISKWGHFYAAYTTTPNFYYYAAYVS DGVIYSVAAYRSQSQRF	79	0.697
4	167	175	VEYIQVYGP	9	0.658

Supplementary Table S5. Summary of the top 10 HDOCK models.

Rank	1	2	3	4	5	6	7	8	9	10
**Docking Score**	-346.11	-339.08	-338.78	-334.86	-321.69	-313.25	-311.13	-310.48	-305.8	-301.35
**Confidence Score**	0.9806	0.9777	0.9776	0.9758	0.9687	0.9632	0.9617	0.9612	0.9575	0.9538
**Ligand rmsd (Å)**	89.27	95.71	71.54	85.32	94.65	95.43	81.4	75.71	72.68	83.43

Note: For HDOCK, when the confidence score is above 0.7, and the docking Score is less than 200, the two molecules would be very likely to bind; when the confidence score is between 0.5 and 0.7, the two molecules would be possible to bind; when the confidence score is below 0.5, the two molecules would be unlikely to bind.

Supplementary Table S6. PDBePISA interface result of candidate TGEV vaccine and TLR4.

Rank	1	2	3	4	5	6	7	8	9	10
**Δ G (kcal/mol)**	-17.4	-15.9	-17.5	-16.6	-10.5	-14.1	-16.5	-11.1	-16.5	-16.9
